# Assessment of recombinant protein production in *E. coli* with Time-Gated Surface Enhanced Raman Spectroscopy (TG-SERS)

**DOI:** 10.1038/s41598-020-59091-3

**Published:** 2020-02-12

**Authors:** Martin Kögler, Jaakko Itkonen, Tapani Viitala, Marco G. Casteleijn

**Affiliations:** 1 VTT Technical Research Centre of Finland, Oulu, Finland; 2 Drug Research Program, Division of Pharmaceutical Biosciences, Faculty of Pharmacy, University of Helsinki, Helsinki, Finland; 3 VTT Technical Research Centre of Finland, Espoo, Finland

**Keywords:** Optics and photonics, Biophysics, Biopolymers in vivo, Protein folding, Proteins

## Abstract

Time-Gated Surface-Enhanced Raman spectroscopy (TG-SERS) was utilized to assess recombinant protein production in *Escherichia coli*. TG-SERS suppressed the fluorescence signal from the biomolecules in the bacteria and the culture media. Characteristic protein signatures at different time points of the cell cultivation were observed and compared to conventional continuous wave (CW)-Raman with SERS. TG-SERS can distinguish discrete features of proteins such as the secondary structures and is therefore indicative of folding or unfolding of the protein. A novel method utilizing nanofibrillar cellulose as a stabilizing agent for nanoparticles and bacterial cells was used for the first time in order to boost the Raman signal, while simultaneously suppressing background signals. We evaluated the expression of *h*CNTF, *h*HspA1, and *h*Hsp27 in complex media using the batch fermentation mode. *HCNTF* was also cultivated using EnBase in a fed-batch like mode. HspA1 expressed poorly due to aggregation problems within the cell, while *h*CNTF expressed in batch mode was correctly folded and protein instabilities were identified in the EnBase cultivation. Time-gated Raman spectroscopy showed to be a powerful tool to evaluate protein production and correct folding within living *E. coli* cells during the cultivation.

## Introduction

*Escherichia coli* is a widely used host organism for the production of recombinant proteins, for example for industrial enzymes^1^ or pharmaceuticals^2,3^. One major limitation when overexpressing heterologous proteins is aggregation or misfolding within the cells which may result in physiological stress to the host organism^1,4^. This stress, activated by the σ_32_-promotor^5,6^, results in the formation of chaperone proteins and proteases^5–7^. The σ_32_-promotor also is activated during the exponential growth phase of *E. coli* and is switched off during the stationary growth phase. Since the σ_32_ protein is unstable and degraded within 4 minutes within the cell, cellular responses are rapid^6^.

For heterologous protein production there is an optimal time-window. The correlation between the growth rate, μ, and the specific protein production rate, q_p_, for induced batch and fed-batch cultures^8^ indicate that a slow growth rate under induced conditions gives little to no product. In addition, there is a limited duration of expression and q_p_, in batch cultures^7^. Several methods probe physiological stress indirectly, either by evaluating bioprocess parameters^7^, or by use of reporter genes under the σ_32_-promotor^9^. These give some insight about the protein aggregation in the cell, and thus ultimately the protein quality^10^. Other techniques aim to measure the amount of protein produced directly from the biomass via a reporter protein^11^ or after sampling^12^. However, there are limited reports on the direct evaluation of the desired product during production in the host cells without removing cells from the culture.

Raman spectroscopy is a promising technique to observe proteins and their secondary structure in a real-time and label free setting without the need for invasive sample handling^13^. It allows to collect accurate qualitative data in the form of a spectrum (fingerprint) of the sample and quantitative data as the intensity of the compounds (vibrations of molecules) in the sample with the use of specific Raman-probes^14^. The quantitative signal should be considered with caution, since any change of the setup, such as focal distance to the sample, stability of the excitation laser source, the detector, as well as the acquisition time have significant influence to the intensity of the Raman peak height and the signal-to-noise ratio. A major advantage of Raman spectroscopy compared to other varieties of spectroscopy, e.g. infrared spectroscopy is that there is very little interference from the vibration of water molecules. This makes Raman spectroscopy an ideal tool for studying liquid samples. However, auto-fluorescence initiated from various biomolecules very often superimposes with the relatively weak Raman signals. There are several methods to suppress interfering fluorescence^15^, and one promising approach is to use time-gated Raman spectroscopy. Surface-Enhanced Raman spectroscopy (SERS) is an approach focusing on the strong enhancement of the Raman signal, which further can minimize the influence of auto-fluorescence^16^. Although Raman setups in the ultraviolet (UV) and infrared (IR) spectral range show less interference of sample-induced auto-fluorescence, the Raman intensity is proportional to the fourth power of the laser excitation frequency and therefore a measurement in UV and IR range may result in less intense Raman emissions and eventual sample degradation^15^. However, excellent protein spectra can be obtained via a thoughtful protocol^17,18^, mainly in low fluorescent matrices.

This contribution used commercially available time-gated Raman spectroscopy^19^, and compared this technology with continuous wave (CW) Raman spectroscopy in order to assess its ability to reduce the high fluorescence signals from complex media and *E. coli* cells. We also compared SERS versus non-enhanced evaluation of the Raman spectra. Finally, we evaluated induced and non-induced *E. coli* batch and fed-batch like cultures at different stages of their growth curve.

## Materials and Methods

### Materials

2x bacto Yeast extract, bacto Tryptone, Phosphate (Thermo Fisher, USA), Glucose (YTPG) medium^20^, Luria-Bertani broth (LB medium);^21^ Growdex nanofibrillar cellulose (NC) was purchased from UPM Biochemicals Oyj (Helsinki, Finland). Disodium hydrogen phosphate was obtained from Fisher Scientific (Hampton, USA), potassium dihydrogen phosphate, calcium chloride, 2-(N-morpholino)ethanesulfonic acid (MES), sodium chloride, glucose, Ethylene diaminetetraacetic acid (EDTA), sodium azide, silver nanoparticles (Ag NPs) − 40 nm particle size (Ag NP; #730807) were obtained from Merck Sigma-Aldrich (Darmstadt, Germany), and potassium chloride was obtained from Honeywell Riedel de Haën (Seelz, Germany). Ultra-pure water, phosphate buffer (buffer A) and MES buffer (buffer B) were prepared as before^22^.

### DNA methods

The pRSETA-HspA1 plasmid was synthesized and codon optimized for *E. coli* (ThermoFisher, USA). The pOPIN-(6HIS)-*h*CNTF plasmid was synthesized as previously described^3^. The pET3a-Hsp27 plasmid was a kind gift of Prof. W. Boelens. The complete DNA sequences of all genes were verified by gene sequencing (GATC, DE) prior to use. *E. coli* NEB5-alpha competent cells (New England Biolabs, USA; chemically competent cells #C2987I) were transformed and used for plasmid propagation^21^. The pRSETA-HspA1 and pET3a-Hsp27 were then transformed separately to *E. coli* strains BL21(DE3) pLYsS and the pOPIN-(6HIS)-*h*CNTF plasmid to *E. coli strain* Rosetta 2 (DE3) pLysS (Novagen, Merck KGaA, Darmstadt, Germany) for protein production.

### Cellular protein expression and protein purification

Expression of HSPA1 and Hsp27 were carried out in BL21(DE3) *E. coli* cells, and *h*CNTF in Rosetta-2 (DE3) *E. coli* cells. First the transformants were grown overnight (o/n) in LB medium with 100 μg/ml ampicillin and 1% (w/v) glucose at 30 °C from previously prepared glycerol stocks according to Sambrook and Russel (2001)^21^. In the case of *h*CNTF in Rosetta-2 *E. coli* cell, 34 μg/ml chloramphenicol (ICN, USA) was added as well. Fifty ml of 2xYPTG medium was inoculated at an OD_600_ of 0.15 at 37 °C from the LB pre-cultures. In addition 2 ml of the *h*CNTF Rosetta-2 *E. coli* o/n culture was used to inoculate 50 ml of EnPressoB medium (EnBase system)^23^ with 100 µg/ml ampicillin and 34 μg/ml chloramphenicol at 30 °C. All cultures were cultivated at 225 rpm (1” amplitude shaker) in high yield flasks^24^ with AirOtop seals (Thomson Instrument Company, CA, USA) to maximize oxygen transfer. Cells in 2xYPTG were induced at OD_600_ = 0.4−0.5 (see Table 1) with 0.4 mM IPTG final concentration and grown for 4 hours (*h*CNTF) or 6 hours (HspA1) after induction. Cells in the EnBase cultures were induced with 0.4 mM IPTG after 24 h and grown for another 24 hours as instructed by the manufacturer. As reference cultures, non-transformed BL21(DE3) *E. coli* cells were cultivated under the same conditions in 2xYPTG and the EnBase system. EnPressoB medium used in the EnBase cultivations was also incubated under the same conditions as the cultivations for the cell dry weight (CDW) measurements to determine the biomass of the EnBase cultures. All samples are summarized in Table 1 and were kept on ice until measured or further processed.

**Table 1:** Shake flask cultivation samples.

Protein	Media	Hours after Induction	OD_600_	Growth Phase
HspA1	2xYTPG	0	0.43	Log
		1^1^	0.89	Log
		2	1.89	Log
		3	3.20	Log
		4^1^	3.65	Log
		5	6.10	Stationary
		6^1^	6.80	Stationary
Hsp27	2xYTPG	0	0.54	Log
		1	1.03	Log
		2	1.32	Log
		3	1.54	Log
		4	1.70	Log
		5	1.91	Log
		6	2.15	Log
*h*CNTF	2xYTPG	−1	0.31	Lag
		0	0.49	Lag
		1^1^	0.82	Log
		2	1.05	Log
		3^1^	1.79	Log
		4^1^	3.32	log
*h*CNTF	EnBase	0^1^	11.7^2^	Log
		24^1^	43.8^2^	Log
—	2xYTPG	4^1,3^	3.04	Log
—	EnBase^4^	0^1^	—	—
		24^1^	—	—

(1) Samples also used for SERS measurements; (2) Cell dry Weight (CDW) was determined and values calculated to OD_600_ as previous described;^24^ (3) These cells acting as negative control were not induced, and this sample was taken 3.5 hours after inoculation (see Fig. 2). The OD_600_ at time-point zero, normalized to time-of-induction of the other samples in Fig. 2 was 0.51; (4) EnBase media without cells was used as a blank for the CDW determination (CDW at 0 hours was 0 and at 24 hours after induction of the *h*CNTF EnBase culture was 12.5 g L^−1^).

### Raman measurements

Raman spectroscopy was performed with both, continuous wave (CW)-Raman and pulsed laser time-gated (TG)- Raman. The TG-Raman instrument (Timegate Instruments Ltd.) was a commercial system to set the benchmark for auto-fluorescence suppression. Both instruments had a laser excitation wavelength of λ_exc_ = 532 nm which is in the fluorescence and Raman maximum response. The commercial CW excitation Raman spectrometer was equipped with a confocal microscope (Nikon Corporation, Tokyo, Japan) with a numerical aperture of 0.22, type alpha 300 RA (WiTec, Ulm, Germany). This was used as the reference for the TG Raman measurements with magnification of 20 × lens and neutral density (ND) filters and laser power of approximately 20 mW (Ophir Nova II laser power meter, Ophir Optronics Solutions Inc., Jerusalem, Israel) at the sample to avoid photo-bleaching. The commercial CW-Raman system was comprised of a conventional thermo-electrically temperature-stabilized charge-coupled device (CCD) detector with optional electron multiplying EM-option (EMCCD) Newton DU970-BV (Andor Technology Ltd, Belfast, Northern Ireland) at operational temperature of −60 °C during the measurements. The spectrometer had a resolution of 4 cm^−1^ full-width-at-half-maximum (FWHM). The TG- Raman instrument was comprised of a non-cooled CMOS SPAD-detector with a 100 ps pulsed Nd:YVO_4_ green laser and adjusted laser power of 20 mW (confirmed with above mentioned laser power meter) at the sample in combination with ND-filters. The system was attached via an adapter to a confocal microscope (Olympus Corporation, Tokyo, Japan) with a magnifying lens of 20 × (NA = 0.4). The TG-Raman spectrometer with a spectral resolution of 10 cm^−1^ (FWHM) had a limited spectral range of 500–1700 cm^−1^. The system was set to cover the temporal decay time of *t* = 0.5–5.5 ns to measure the Raman and fluorescence signal while being able to separate both signals from each other. A detailed description of the time-gating principle used in the Timegate Instruments Ltd. device can be found elsewhere^15,25,26^.

### Measurement procedure and spectral data processing

**Figure 1:**
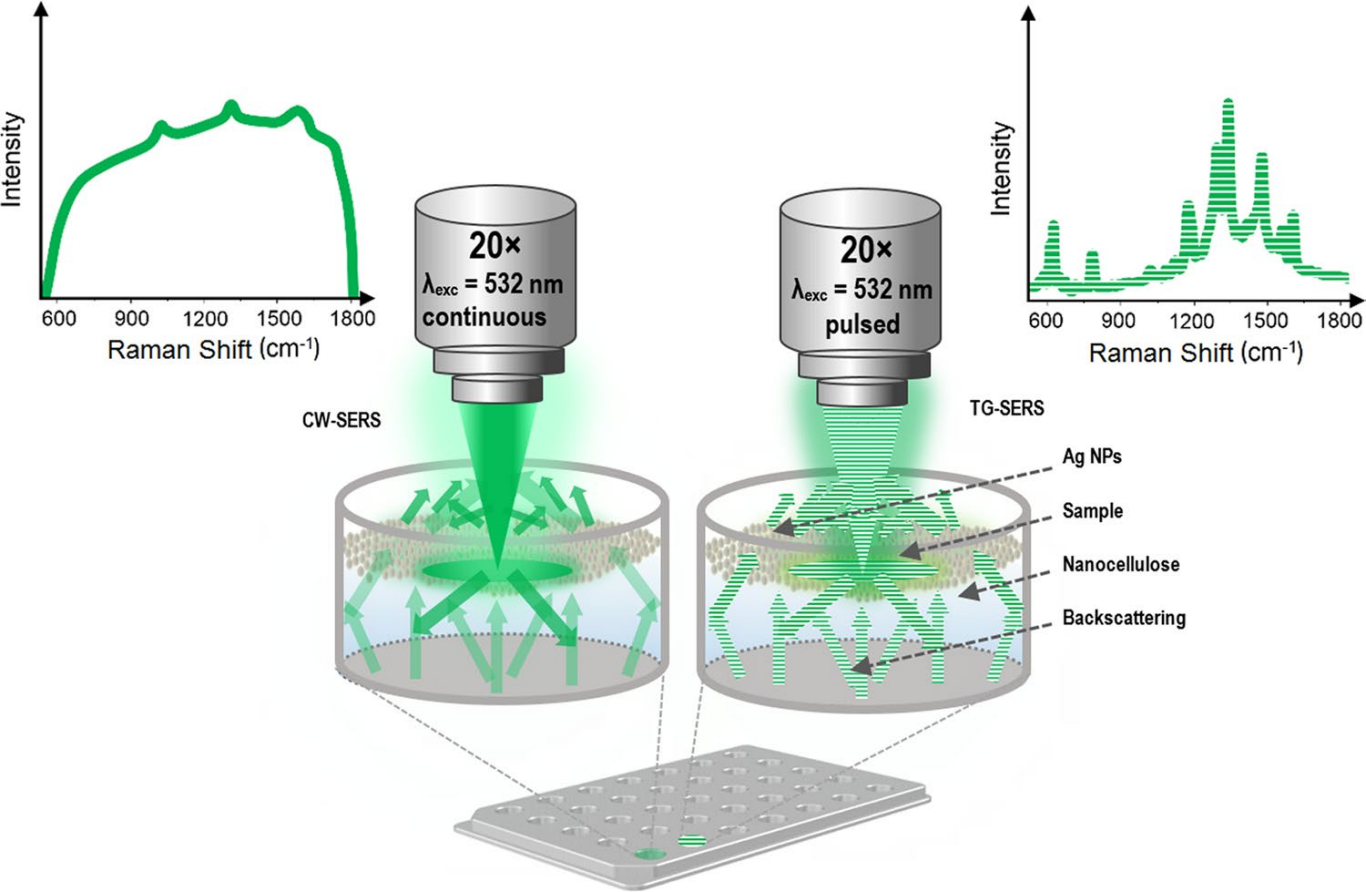
Experimental Raman microscope setup with conventional CW-SERS (left side with constant green) and pulsed laser TG-SERS (right side with dashed green line) both at λ_exc_ = 532 nm using Ag NPs on top of a nanofibrillar cellulose (NC) soft bed together with the media sample filled into the aluminium well plate.

The commercial Ag NPs stock solution was centrifuged (Eppendorf 5804 R, rotor FA–45–6–30) at 4500 rpm for 4 min., followed by the removal of the supernatant which reached a final concentration of around 0.06 g L^−1^. The experimental Raman setups including the assay (bottom-up) of NC, Ag NPs, and sample is depicted in Fig. 1. Preceding the actual Raman/SERS measurements, each well of an in-house made anodized aluminum microwell plate (Fig. S1) with a total volume of 90 µL/well (Fig. 1) was filled with 25 µL NC. Layered on top was 25 µL Ag NPs before 25 µL of the sample (Table 1) was added, with a total volume of 75 µl. Each sample was prepared fresh for each microwell (i.e. the layering of NC, Ag NPs and cells) with the same settings prior to each Raman/SERS measurement, both in CW and time-gated settings. The final concentration of the cells was approximately 4 * 10^8^ cells/ml (*h*CNTF 2, 4, and 24 hours, and HspA1 6 hours after induction). The final concentration for *h*CNTF and HspA1 1 hour after induction was approximately 2.4 * 10^8^ cells/ml, considering the volume of NC and Ag NP solutions. The aluminum has proven to not interfere with the measurements^27,28^. In addition, due to the relatively low intensity of background from complex media from the *E. coli* cell samples with NC and Ag NPs this contribution was not subtracted from final spectra (Fig. S6).

Spectral data processing was performed with OriginPro (V. 2016b and 2018b, OriginLab, Northampton, MA, USA). The data was normalized in an intensity interval between 0 and 1 and plotted with an offset for better presentation, except for Figs. 4 and 6 (bottom row) where Raman intensities from two TG-Raman measurements were compared with each other. Prior detailed spectral analysis and comparison, all TG-Raman data was pre-processed with the TG-Raman instrument spectral processing tool (Timegate Instruments Oy, Oulu, Finland). Prior to the measurements, both Raman spectrometers were wavelength calibrated and the excitation lasers sources operated normally.

**Figure 2:**
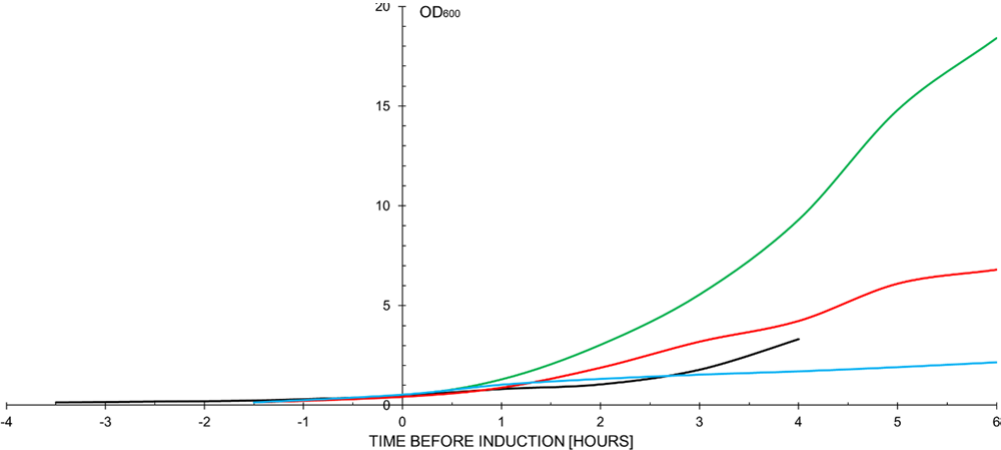
Shake flask cultivations of *E. coli* BL21(DE3) with no plasmid (green), BL21(DE3) pRSETA-HspA1 (red), BL21(DE3) pET3a-Hsp27 (blue), Rosetta-2 pOPINF-*h*CNTF-1 (black) cells in 2x YTPG medium. EnBase system cultivations of Rosetta-2 pOPINF-*h*CNTF-2 are not shown (cell dry weights are listed in Table 1). The figure was created with Microsoft Excel version 16.0.4927.1000; https://products.office.com/en/excel).

**Figure 3:**
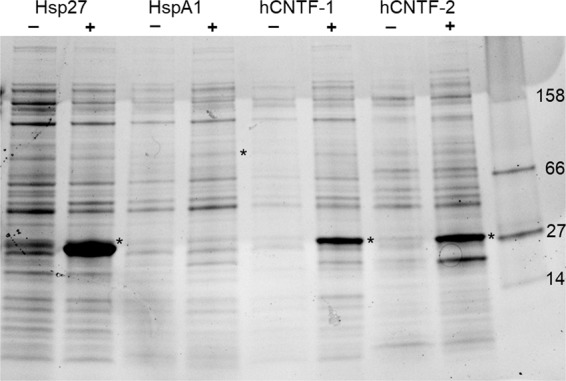
Recombinant protein expression of Hsp27, HspA1, and *h*CNTF in a batch fermentation process with 2xYTPG rich medium (*h*CNTF-1) for 4 hours, and *h*CNTF in continuous fermentation process with EnBase medium^23^ (*h*CNTF-2) for 24 hours. All fermentations were done in high yield flasks^45^ at 30 °C and 225 rpm (1” amplitude). The SDS-PAGE compared the amount of protein per cell before induction (−) and after induction with IPTG (+). The prepared target protein is indicated by an asterisk.

## Results

### Protein expression

Heterologous protein production performed in shake flasks, in batch mode using *E. coli* expression strains, were monitored for their cell-growth by sampling every hour and measuring the optical density with a spectrophotometer at λ_abs_ = 600 nm (OD_600_). Absorbance levels of OD_600_ > 0.5 were diluted to avoid a bias in the measurements (Table 1).

The impact of heterologous protein production after induction on the growth of *E. coli* is clearly observable (Figs. 2 and 3). *E. coli* cells that did not contain an expression plasmid obtained a much higher final cell-density than induced cultures. Rosetta-2 cells expressing *h*CNTF grew slower and were induced later than the other samples, hence the relative shorter expression time of 4 hours versus 6 hours in BL21(DE3) cells.

**Figure 4:**
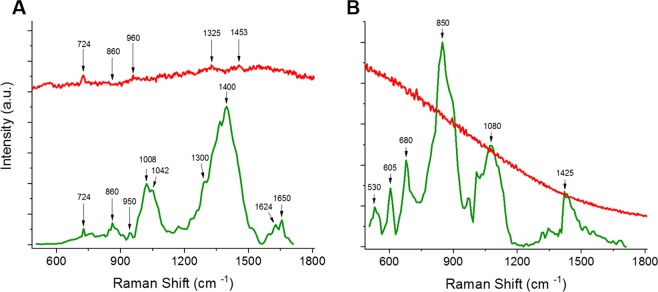
Comparison of continuous Raman (red – CW-SERS) and time-gated Raman with SERS (green – TG-SERS) at λ_exc_ = 532 nm (**A**) of *E. coli* cells in media expressing *h*CNTF (1 hour after induction) and (**B**) eGFP in buffer. The image was created with OriginPro (V. 2016b and 2018b; https://www.originlab.com/index.aspx?go=Products/Origin).

EnBase system cultures mimicked a fed-batch cultivation^23^ and therefore reached a much higher optical density. They were induced at OD_600_ = 11.7 after 24 hours of growth and harvested at OD_600_ = 43.8 (Table 1). As can be seen in Fig. 3, Hsp27 and *h*CNTF proteins were produced, while the levels of HspA1 were barely visible on the SDS-PAGE gel after Coomassie staining. From previous experience we know that most of the HspA1 protein ends up in inclusion bodies (IB) due to misfolding under these conditions (data not shown). Human heat shock proteins are known for their instability during heterologous expression in *E. coli* fermentations^29,30^. Unexpectedly, *h*CNTF expressed with the EnBase system showed 2 bands after induction, while *h*CNTF produced under similar conditions as before^3^ showed one product. Since we had stored previous batches of *h*CNTF in conditions where protein was denaturing, i.e. before we optimized the storage buffer^22^, we chose HspA1 and both *h*CNTF samples for further evaluation with Time-Gated Raman spectroscopy as representative samples of 3 types of fermentations: (a) poorly expressed protein (HspA1), well expressed protein (*h*CNTF batch mode), and a degrading protein (*h*CNTF in fed-batch like mode). CW-Raman spectra of Hsp27 can be found in the supplementary data (Figure S3).

### Time-Gated Raman Spectroscopy in liquid samples

The purpose of our experimental set-up was to measure directly from expression culture, without removal of the expression media or drying the samples. Therefore, we used a relatively large volume of 75 µl with a measured laser power of 20 mW to assure minimal heating of the sample. The fluorescence of the samples is a major problem when measuring at λ_exc_ = 532 nm as can be seen in Fig. 4. Figure 4A shows the comparison between Continuous Wave (CW)-SERS (red curve) and TG-SERS (green curve) measurements of *h*CNTF 1 hour after induction under the same experimental set-up as depicted in Fig. 1. For clarity, the intensities have been standardized in an interval between 0 and 1 and off-set separated. These results clearly demonstrates the ability of the TG-Raman technique to suppress interfering fluorescence. Even when Ag NPs are added to CW-Raman measurements, the signals are barely distinguishable from the background signals (Figs. 4 and S3). When looking at the overall spectral range (200–3000 cm^−1^) of the CW-SERS measurements (Figure S2) the level of fluorescence appears as a broad bump. However, some weak spectral peaks are faintly visible (724, 850–860, 950–960, 1330 as well as 1450 cm^−1^) which correspond to the more clearly identifiable TG-Raman spectra. Figure 4B represents a measurement of enhanced Green Fluorescent Protein (eGFP) sample with CW-Raman (red) and TG-Raman (green) with even stronger fluorescence background in the conventional CW-Raman setup. Furthermore, even though the CW-Raman system has an excellent detector, it saturated very rapidly, i.e. under 10 seconds acquisition time, and no obvious Raman-protein spectrum was observed. In comparison, the TG-Raman spectrometer at even at over 1 million photon-counts obtained a typical Raman protein spectrum (Fig. 4) in line with peaks at intensities described before (Table 2).

**Figure 5:**
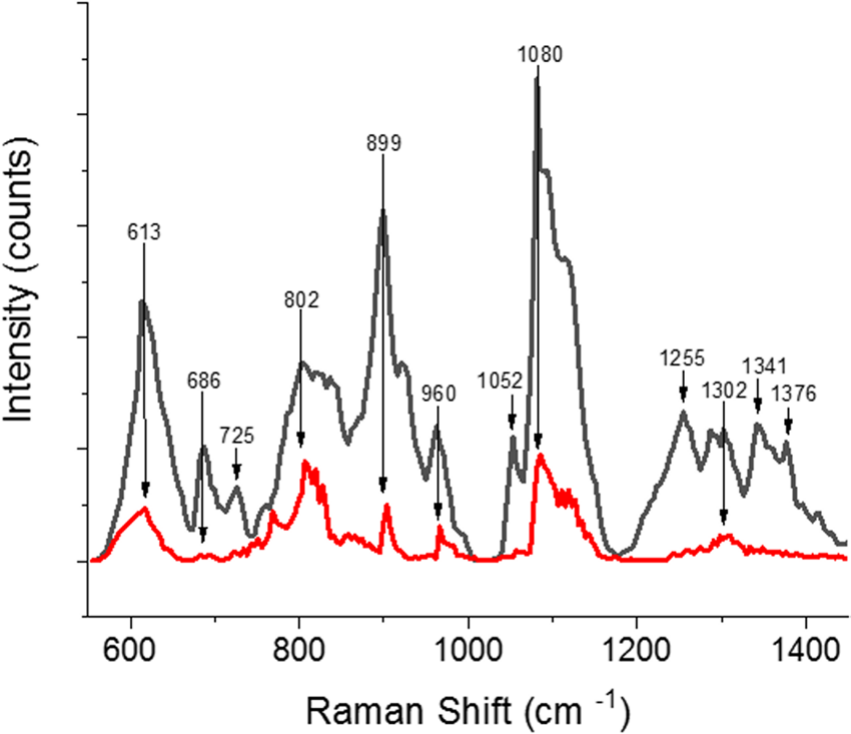
Suppression of NC background with TG-SERS (red curve) and without background suppression using only NC (black curve). The image was created with OriginPro (V. 2016b and 2018b; https://www.originlab.com/index.aspx?go=Products/Origin).

**Table 2:** Tentative assignments of Raman/SERS bands.

Measured Raman/SERS bands [cm^−1^]	Literature reference Raman/SERS bands [cm^−1^]	Tentative assignments (interpretation of Raman/SERS bands)	Origin/Category	reference
**530**	510–550	S-S bond stretching	Proteins containing S-S bridges	^18,46^
**550–580**	560–580	Ring and CH-deformation	Carbohydrate, yeast and growth medium	^27^
**613**	620	Aromatic amino acids	Phenylalanine	^46^
**710–735**	732	DNA /CH_2_ rocking	Adenine or cAMP, growth medium	^27^
**820–860**	830–860	Aromatic amino acids, NC minor influence	Tyrosine and/or NC	^13,18,46^
**918–925**	924–943	N−C_α_−C stretch	Valine	^18^
**942–950**	957–964	C = C deformation	DNA (guanine)	^27,47^
**1008–1030**	1000, 1030	Aromatic amino acids, NC minor influence	Phenylalanine and/or NC	this work^46,48^
**1050**	1033	C−H plane bending of aromatic compounds	Phenylalanine, tryptophan, tyrosine	this work^49^,
**1130–1195**	1134–1160	C−N and C−C stretch	Carbohydrate in medium	^27^
**1170–1195**	1170–1200	Aromatic amino acids	Tyrosine	^48^
**1232–1242**	1232–1250	N−H and C−H bend	Amide III (β-sheet)	^18,50^
**1295–1336**	1300−1345	N−H and C−H bend	Amide III (α-helix)	^13,50^
**1365–1367**	1320–1340	DNA, Nucleotide	Adenine (AMP)	^27^
**1398–1405**	1390–1398	Symmetric deformation of CH_3_ or overtone of Amide V	Amide V or Peptide, growth medium	^13,51^
**1423**	1420–1480	DNA or CH_2_ deformation	Adenine, Guanine	^47^
**1450**	1444–1450	CH_2_ deformation	Lipid	^18,48^
**1505–1515**	—	CH_2_ deformation	Carbohydrate in growth medium	this work
**1520–1550**	1550	N–H bend and C–N stretch	Amide II	^46^
**1590**	1582–1590	Aromatic amino acids	Tryptophan or Tyrosine	^46^
**1605**	1609	Aromatic amino acids	Phenylalanine or Tyrosine	this work^46^
**1624–1627**	1617	Aromatic amino acids or part of C = C stretch	Tryptophan or Tyrosine	^18,47^
**1650–1657**	1660–1677	C = O stretch	Amide I	^18,46^

To ensure that the time-gated laser would sample enough *E. coli* cells in the measuring area under the objective and avoid cell movements due to convection currents, we made use of nanofibrillar cellulose (NC) to immobilize the cells (Fig. 1). NC has been shown to be an excellent inert matrix for cells^31^, however since cellulose has a rather strong Raman spectrum overlapping with protein peaks, we used Ag NPs to suppress the Raman signals from the underlying matrix (Fig. 5). As can clearly be seen, even at relative low concentrations of 0.06 g L^–1^of AgNPs, most of the cellulose spectra were suppressed, while citric acid present in the Ag NPs solution as preservative is not observed in the final NC, Ag NPs, and cell samples (Figure S5).

**Figure 6:**
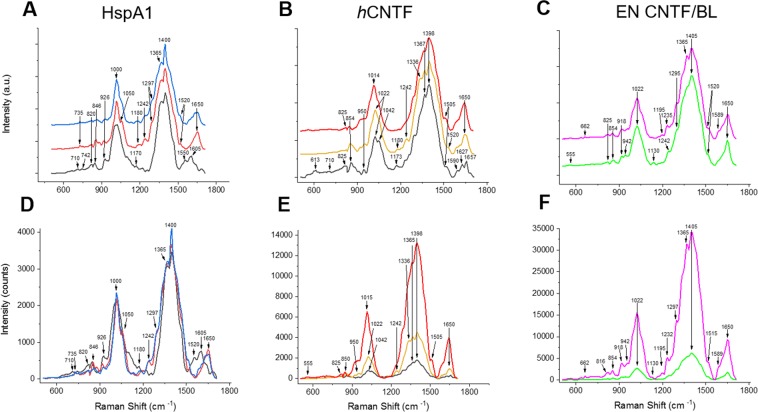
Overlapping TG-SERS spectra at different time points. (**A**) HspA1 at 1 h (black), 4 h (red) and 6 h (blue), (**B**) *h*CNTF at 1 h (black), 3 h (yellow) and 4 h (red) and (**C**) EnBase cultivated (EN): *h*CNTF (magenta) compared to blank EN BL (green) after 24 h. (**D**–**F**) Shows corresponding Raman intensities (detector counts) and A-C the normalized arbitrary Raman intensities, respectively. The image was created with OriginPro (V. 2016b and 2018b; https://www.originlab.com/index.aspx?go=Products/Origin).

### Evaluation of protein production in *E. coli* cells with Raman Spectroscopy

Living *E. coli* cells were evaluated with CW-Raman spectroscopy and TG-Raman spectroscopy with and without SERS enhancement. The results for HspA1 and two different *h*CNTF cultivations are summarized in Fig. 6. Due to the strong background fluorescence, not much can be seen in the CW-Raman/SERS spectra (Figs. 4A, 6, S2 and S3). Hence, in order to evaluate the protein quality within the cells normalized TG-Raman spectra were compared (Fig. 6A–C). In addition, the changes of the real intensities of the spectra (Fig. 6D–F) for the same amount of cells indicate the protein production rate and identify additional quality parameters. All cultivation data shown in Fig. 6 have been scaled within the same spectral range in order to simplify comparison between the different spectra.

In general, three strong spectral peaks were very distinctive in HspA1 and the *h*CNTF cultivations, namely around 1000, 1400 and 1650 cm^−1^, but their intensities depended on the stage of the cultivation. In particular it appears that the phenylalanine peak around 1000 cm^−1^ is broad and seems to be a convolution of another peak at around 1050 cm^−1^ (cp. Table 2). In addition, the peaks around 1400 cm^−1^ in all cultivations at different time points showed a double or triple peak. This might indicate either a symmetric deformation of CH_3_ or an overtone of Amide V was present. It also appears it was the strongest and widest peak increasing as a function of time. Even though, the increase of the peaks around 1400 cm^−1^ are clearly due to increase of protein per cell (Fig. 6E,F), quality parameters are harder to identify, since these peaks in the normalized spectra of Fig. 6A–C may also be present in the cultivation media (Figure S6).

Small differences can be identified at the different time points, i.e. HspA1 (first row) after 1 h of induction shows an elevated background with DNA signatures at 710 and 742 cm^−1^ that is not present in later stages of the cultivation. During the exponential growth phase approximately 3% of the cell dry weight consists of DNA^32^, however growth rates tend to be lower under induced conditions^8^ and DNA amounts per cell are lower at lower growth rates^33^. The peaks around 850 cm^−1^ are weak which may indicate that NC as “soft bed” for the assay (Fig. 5) does not interfere while observing the protein Raman spectra. We speculate that the peak can be attributed to tyrosine, due to the shift of the maximum intensity from 846 cm^−1^ to 862 cm^−1^ in the HspA1 cultivation (Fig. 6A), which indicates changes in local interactions within the protein. We do not observe this effect in the *h*CNTF cultivation, thus we attribute this shift due to protein aggregation or the formation of IBs.

Larger differences can be observed when comparing the intensity values (Fig. 6D–F). The differences of the HspA1 and *h*CNTF batch cultivations after 1 hour of induction region are rather small, but shift clearly in the later time points. The spectral data reflect the amount of protein produced per cell, which is evident when comparing expression levels in the SDS-PAGE gel (Fig. 3). The total amount of protein in a non-induced culture compared to an induced culture after 24 hours of protein expression in the EnBase system *h*CNTF (EN *h*CNTF; Fig. 6F) is higher than in the batch phase protein expression of *h*CNTF depicted in Fig. 6B,E. The amide III peaks at 1242 cm^−1^ and around 1300 cm^−1^ are clearly seen as shoulders of the main peak around 1400 cm^−1^ of cells under induced conditions. The pronounced amide I peak is increasing as well during the *h*CNTF cultivations. Clearly visible is the difference between HspA1 and *h*CNTF cultivations. HspA1 shows peaks at 1520–1680 cm^−1^ with traces of Amide II and aromatic structures at 1550 and 1605 cm^−1^, while the shoulder at ~1590 cm^−1^ of the α-helical *h*CNTF is indicative of aromatic modes^34^.

Overall, the time-gated Raman spectra showed similar spectra compared to cell or protein spectra provided by the references listed in Table 2.

## Discussion

Biotechnology and synthetic biology are considered of increasing importance to provide solutions for the sustainable production of food, medicine, vaccines, industrial enzymes, chemicals, and components for materials such as bioplastics. The EU lists industrial biotechnology as one of the key enabling technologies (KET) for our future. In comparison to other technologies, biotechnological production is still complex and undefined, and product quality is not easy to control. Therefore, new accurate methods are needed to better understand the manufacturing of products. As an analogy, the industrial revolution was driven by mass production of standardized parts. In a similar way to truly transform the biotechnology industry for precision manufacturing, the lack of tools to directly monitor the intermediate products during manufacturing within the living cells must be addressed.

In this study, we showed for the first time the use of TG-SERS for the direct evaluation of recombinant protein production within living bacterial cells and compared this with CW-SERS. The use of TG-SERS significantly reduced the interference of the background fluorescence. We utilized a novel method to stabilize our cultivation media samples for the measurements on top of NC. Furthermore, we utilized the effect of the Raman enhancement with SERS to provide a more stable measurement surrounding in order to compare measurements from different time points in the cultivations. We could clearly identify differences in the cultures without the need to dry the samples or break the cells.

HspA1 consists of two domains: (a) the substrate binding domain, which consists mainly of β-sheets and one alpha-helix^35^ and (b) the ATP binding domain, which consists of both α-helices and β-sheets^36^. When comparing the HspA1 spectra with the spectra of the helical protein *h*CNTF (Fig. 6), besides the difference of the amide I peaks^34^, the valine peak at 926 cm^−1^ is prominent; HspA1 contains 7% of valine. One other prominent feature of human HSPA1 Raman spectra is the shoulder of the broad 1014 cm^−1^ peak at 1050 cm^−1^, which could be assigned to lipids. However, it was shown by Höhl *et al*. (2019) that a distinct peak at 1100 cm^−1^ after data post-processing could be contributed to pure HSPA1 and not to Hsp90 under the same conditions^37^. Therefore, we speculate that the shoulder at 1050 cm^−1^ could be due to the δ_C-H_ plane bending of aromatic side chains, such as phenylalanine, tryptophan and/or tyrosine (Table 2).

The preparation of soluble and active HspA1 is often limited by misfolding during expression, which in *E. coli* can lead to the formation of IBs^29,38^. The misfolding of HspA1 is evident when observing the shift of the amide I peak from 1680 to 1650 cm^−1^ between 1 to 4 hours of expression. This shift is due to the formation of amyloid structures and additional β-sheets, and is linked to protein aggregation^39,40^.

Another issue of HSPA1 expression in BL21(DE3) strains is the interaction of HSPA1 with native *E. coli* GAPDH, which may result in a slower growth^41^. Briand *et al*. described the leakage of the hHSPA1 gene and the interaction with GAPDH, which resulted in the auto-induction of hHSP70 production due to the inactivation of the LacI gene in pET plasmids. However, the expression plasmid pRSETA we utilized in our study did not contain the LacI gene. The limited expression rate of HspA1 in our experiments is evident when comparing the SDS-PAGE gel (Fig. 3, lanes 3–4) with the relative small increase of the phenylalanine peak (1014–1050 cm^−1^) in Fig. 6D after four and six hours induction. Low growth rates are known to have an effect on protein production rates in *E. coli*^8^.

Human CNTF is a small, fully α-helical protein^42,43^. Throughout the expression, the amide I peak at 1650 cm^−1^ clearly has the characteristic shape originating from an α-helical structure with its maximum at 1650 cm^−1^ (Table 2) and the aromatic modes or amide II peak at 1598 cm^−1^^34^. During the expression, the phenylalanine peak shifts to a lower wavenumber of 1014–1022 cm^−1^, presumably due to folding of the protein. This behavior was not observed in the HspA1 cultivation. The most abundant amino acid in *h*CNTF is leucine (13%), which may be a reason why the maximum of the broad peak at ~1400, mainly due to the culture media (Figure S6), is shifted slightly to a lower wavelength, since leucine does show a peak at a maximum of 1395 cm^−1^^44^.

**Figure 7:**
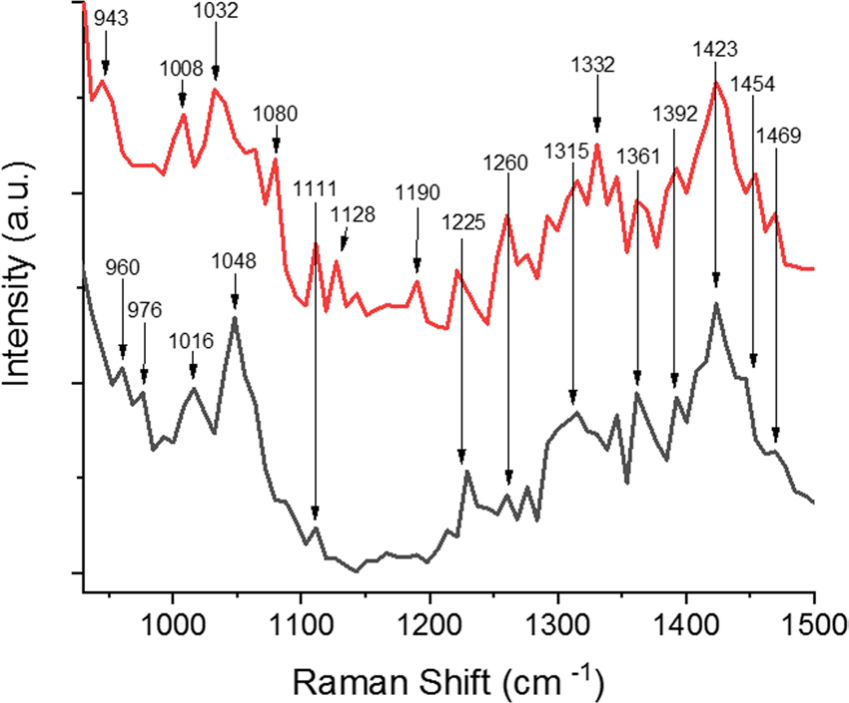
*h*CNTF old batch (red curve) and new batch (black curve). The image was created with OriginPro (V. 2016b and 2018b; https://www.originlab.com/index.aspx?go=Products/Origin).

In order to explain the differences in *h*CNTF spectra between the batch cultivation and the EnBase fed-batch like cultivation (Fig. 6B,C,E,F) we further evaluated the difference between a new and an old batch of *h*CNTF cultivation with TG-SERS. These samples were purified and stored in buffer^3^ or directly measured after purification by using an optimized buffer for improved stability^22^. Figure 7 shows the results within a spectral range of 900–1500 cm^−1^. Differences at 960, 976, around 1000 and 1080, 1128, 1190, 1315, 1332 cm^−1^ are noticeable also after repeated measurements. However, shape and structure of the spectra remains practically the same.

We compared the expression of *h*CNTF in the SDS-PAGE gel (Fig. 3; lanes 6 and 8) with the spectra observed in Figs. 6 and 7, and it is evident that *h*CNTF cultivated with the EnBase system shows signs of degradation at the end of the cultivation. The additional strong band at ~ 23 kDa in Fig. 7 (lane 8) is weak in the batch cultivation (lane 6) indicating protein degradation. In the Raman spectra obtained during the cultivations (Fig. 6), we observed the following differences in the EnBase cultivation compared to the *h*CNTF batch cultivation: (1) a minor phenylalanine peak at 662 cm^−1^, (2) a shift of the tyrosine peak to 816 cm^−1^, (3) a distinct valine peak at 918 cm^−1^, (4) minor peaks at 1130 and 1195 cm^−1^, and (5) indication of β-sheet formation due to the rise of a peak at 1332 cm^−1^. Thus, the changes in the protein environment are seen via the aromatic amino acids. In addition, degraded/aggregated *h*CNTF in buffer compared to newly prepared *h*CNTF shows two small, but distinct peaks at ~1130 and ~1195 cm^−1^. These peaks are indicative of protein degradation during protein expression in the EnBase system. Taken together, we found several indications of protein degradation of *h*CNTF in the time-gated-Raman spectra during the EnBase system cultivation.

## Conclusion

Time-gated Raman spectroscopy showed to be an advantageous spectroscopic tool to evaluate protein expression conditions during *E. coli* fermentations. Despite the longer sampling time compared to CW-Raman spectroscopy, the suppression of highly fluorescent signal is crucial to obtain meaningful Raman signals when measuring directly from living *E. coli* cells within complex cultivation media. Time-gated Raman spectroscopy can identify the increase of protein levels in the cell after induction and specific peaks indicative of protein quality, e.g. protein aggregation or degradation. More specifically, we showed with Raman spectroscopy measurements that protein production levels were low and that the protein was aggregating during the cultivation of HspA1. Regarding the cultivation of *h*CNFT we identified specific quality parameters indicative of correct protein folding. Therefore, time-gated Raman spectroscopy is a powerful tool to directly monitor the intermediate production steps during manufacturing with living cells needed for future precision manufacturing of proteins.

## Availability of data and materials

The datasets supporting the conclusions of this article are included within the article and the supplementary data file.

## Supplementary information

Supplementary Information.
