# Supplementary material for: Assessment of recombinant protein production in E. coli with Time-Gated Surface Enhanced Raman Spectroscopy (TG-SERS)
Source: Sci Rep. 2020 Feb 12;10:2472. doi: 10.1038/s41598-020-59091-3 (PMC7015922; doi:10.1038/s41598-020-59091-3)
Supplement: Supplementary file 1 — Supplementary Information. [file 41598_2020_59091_MOESM1_ESM.pdf]

Assessment of recombinant protein production in *E. coli* with Time-Gated Surface Enhanced Raman Spectroscopy (TG-SERS)

Martin Kögler[1], Jaakko Itkonen[2], Tapani Viitala[2], Marco G. Casteleijn[#2,3].

[1]VTT Technical Research Centre of Finland, Oulu, Finland. [2]Drug Research Program, Division of Pharmaceutical Biosciences, Faculty of Pharmacy, University of Helsinki, Finland. [3]VTT Technical Research Centre of Finland, Espoo, Finland

[#]Corresponding author: Marco G. Casteleijn, VTT Technical Research Centre of Finland, Espoo, Finland, marco.casteleijn@vtt.fi

Supplementary Data:

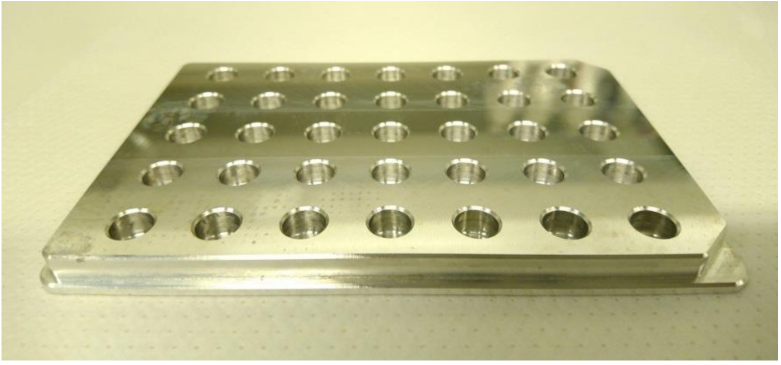

Figure S1. The aluminum 35 well-plate used for Raman measurements.

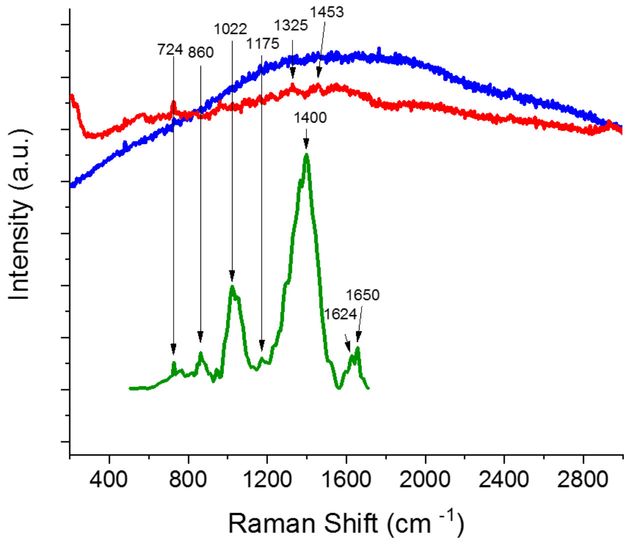

Figure S2. *E. coli* cells in media measured with CW-Raman (blue) and CW-SERS (red) compared to TG-SERS (green). In analogy to Figure 4A, the spectra are shown in the spectral range from 200 – 3000 cm^{-1} N.B. The TG Raman spectrum can only be displayed in a limited spectral range 500 – 1700 cm^{-1}. The image was created with OriginPro (V. 2016b and 2018b; https://www.originlab.com/index.aspx?go=Products/Origin).

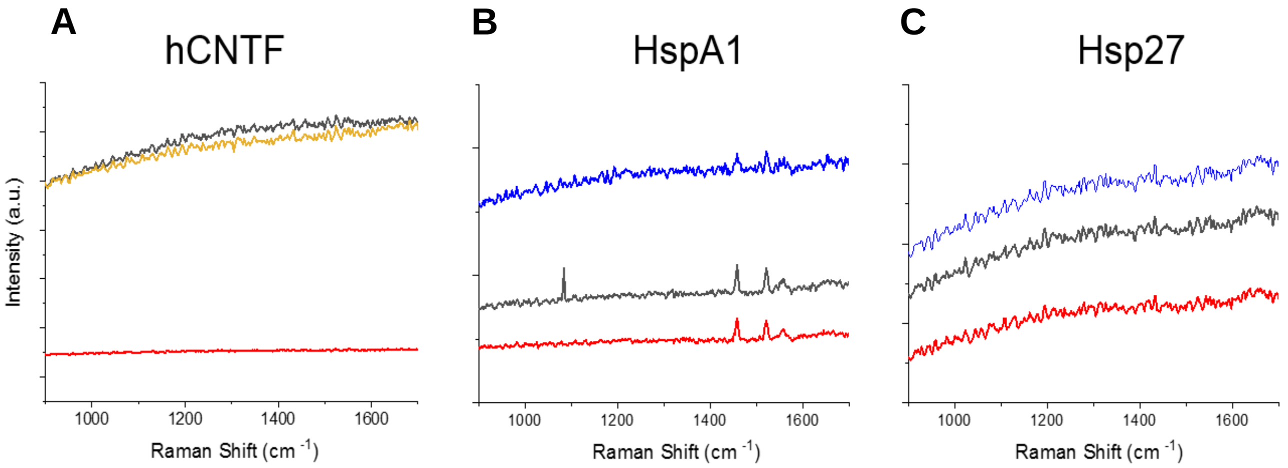

Figure S3. CW-SERS from time points of A) 1h (black), 3h (yellow), 4h (red) expression of *h*CNTF in *E. coli*, B) 1h (black), 4h (red), 6h (blue) expression of HspA1 in *E. coli,* and C) 1h (black), 4h (red), 6h (blue) expression of Hsp27 respectively using same color code as in figure 6. The image was created with OriginPro (V. 2016b and 2018b; https://www.originlab.com/index.aspx?go=Products/Origin).

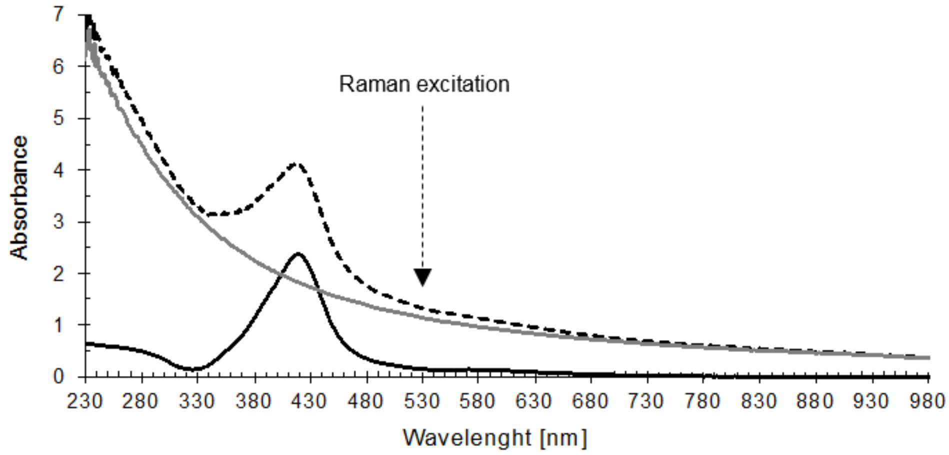

Figure S4. UV-VIS spectra of silver nanoparticles - visible and ultraviolet spectrum of Ag NPs (black), NC (grey), and combined Ag NPs + NC (dashed), all at same concentrations as utilized in the Raman measurements at λ_{exc} = 532 nm. The figure was created with Microsoft Excel version 16.0.4927.1000; https://products.office.com/en/excel).

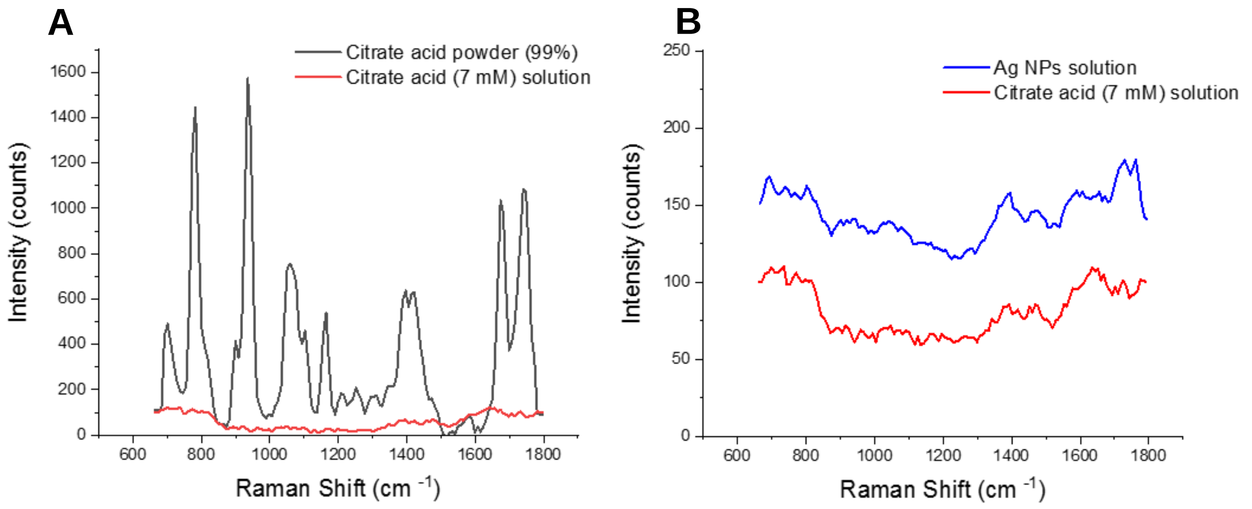

Figure S5. The influence of citric acid present in Ag NPs solution on the TG-Raman measurements – A) citrate acid powder (99%; black) compared to the actual used concentration of approx. 7 mM citrate acid in solution, and B) Comparison of TG-Raman spectra of citrate acid solution (red) without Ag NPs particles and Ag NPs in solution, containing citric acid as indicated by the manufacturer (blue). The latter were mainly used to suppress the interfering spectra originating from nanocellulose (cp. materials and methods section and Fig. 5 of the manuscript). The image was created with OriginPro (V. 2016b and 2018b; https://www.originlab.com/index.aspx?go=Products/Origin).

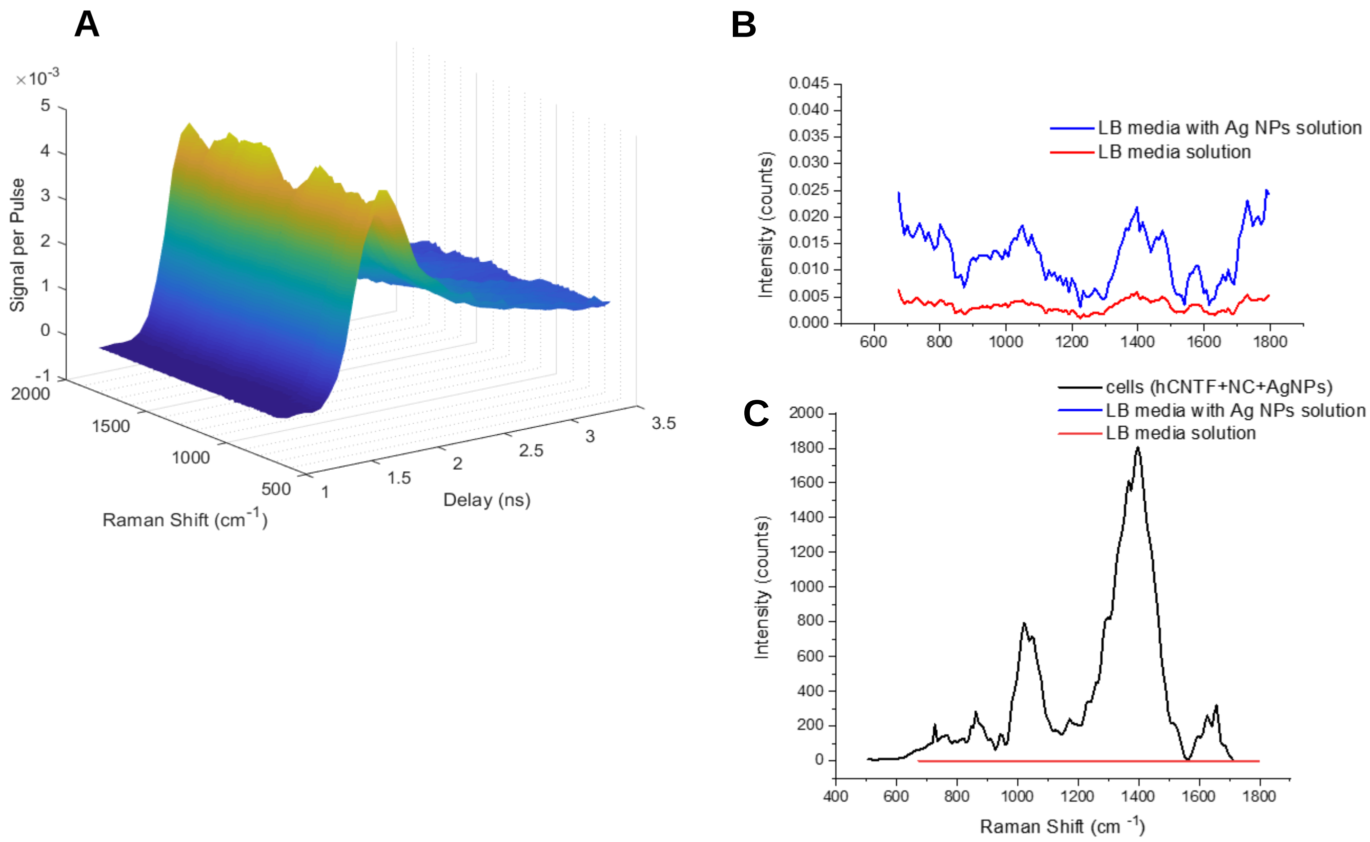

Figure S6. The influence of complex media and its contribution to the TG-Raman measurements – A) three-dimensional accumulated TG-Raman RAW data (interval shortened between 1 and 3.5 ns for better representation) of LB medium with Ag NPs, B) Extracted two-dimensional spectra of LB medium with (blue) and without (red) Ag NPs, and C) TG-Raman spectra of LB medium with and without Ag NPs (Blue and Red lines respectively are overlapped due to the scale) in comparison to living *E. coli* cell expressing *h*CNTF in complex medium (black). Figure A was created using Timegate Instruments Ltd. shsgui - Spectral Processing Tool version 0.963 (https://www.timegate.com/timegated-technology) and figures 6B and C were created with OriginPro (V. 2016b and 2018b; https://www.originlab.com/index.aspx?go=Products/Origin).

Uncropped images

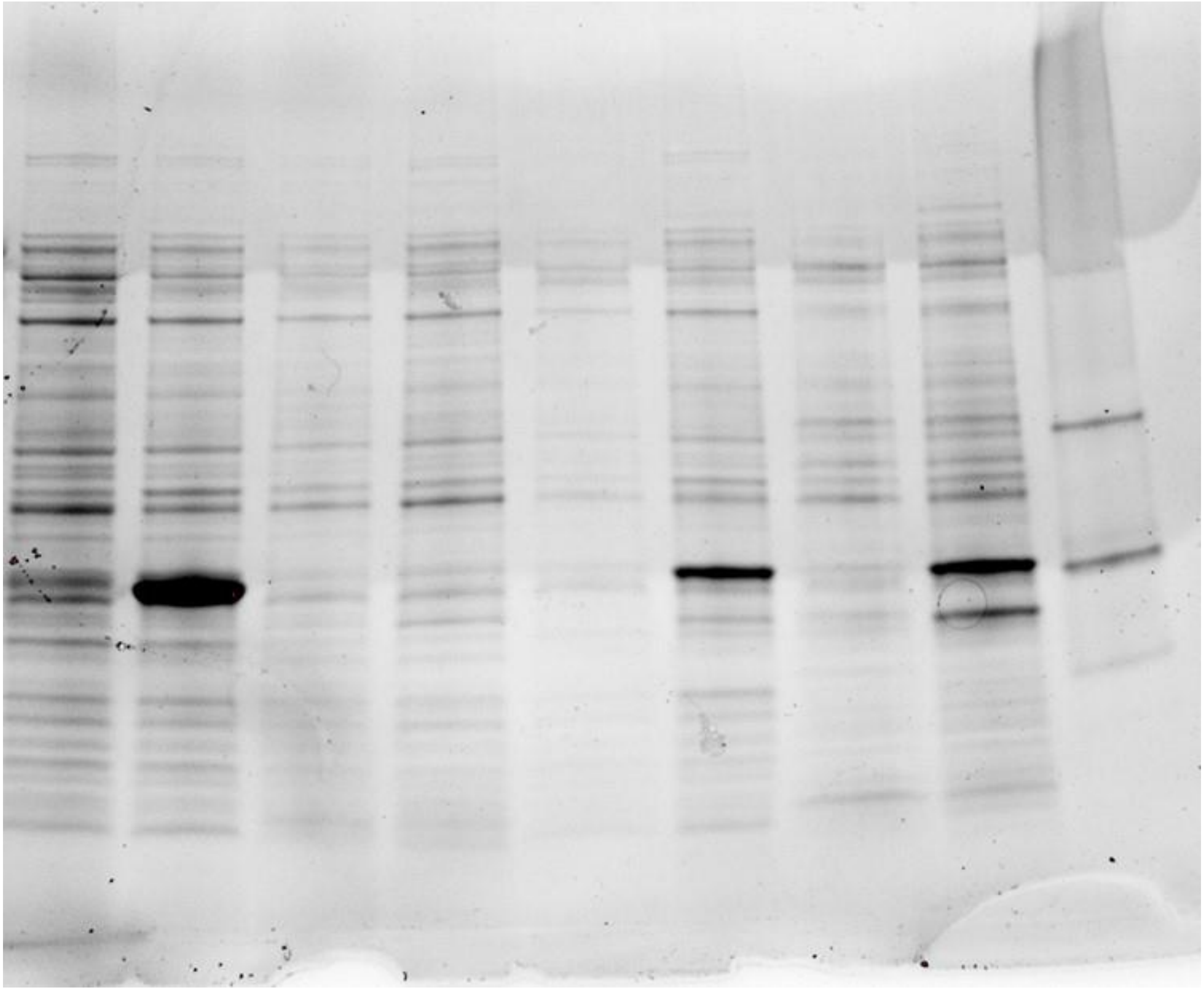

Figure S7. Uncropped SDS-PAGE gel as used for figure 3 in the main text.
